# PADLOC: a web server for the identification of antiviral defence systems in microbial genomes

**DOI:** 10.1093/nar/gkac400

**Published:** 2022-05-25

**Authors:** Leighton J Payne, Sean Meaden, Mario R Mestre, Chris Palmer, Nicolás Toro, Peter C Fineran, Simon A Jackson

**Affiliations:** Department of Microbiology and Immunology, University of Otago, Dunedin, New Zealand; Biosciences, University of Exeter, Penryn, UK; Independent Researcher, Spain; Information Technology Services Research and Teaching Group, University of Otago, Dunedin, New Zealand; Department of Soil Microbiology and Symbiotic Systems, Estación Experimental del Zaidín, Consejo Superior de Investigaciones Científicas, Structure, Dynamics and Function of Rhizobacterial Genomes, Grupo de Ecología Genética de la Rizosfera, Granada, Spain; Department of Microbiology and Immunology, University of Otago, Dunedin, New Zealand; Genetics Otago, University of Otago, Dunedin, New Zealand; Bioprotection Aotearoa, University of Otago, Dunedin, New Zealand; Maurice Wilkins Centre for Molecular Biodiscovery, University of Otago, Dunedin, New Zealand; Department of Microbiology and Immunology, University of Otago, Dunedin, New Zealand; Genetics Otago, University of Otago, Dunedin, New Zealand; Bioprotection Aotearoa, University of Otago, Dunedin, New Zealand; Maurice Wilkins Centre for Molecular Biodiscovery, University of Otago, Dunedin, New Zealand

## Abstract

Most bacteria and archaea possess multiple antiviral defence systems that protect against infection by phages, archaeal viruses and mobile genetic elements. Our understanding of the diversity of defence systems has increased greatly in the last few years, and many more systems likely await discovery. To identify defence-related genes, we recently developed the Prokaryotic Antiviral Defence LOCator (PADLOC) bioinformatics tool. To increase the accessibility of PADLOC, we describe here the PADLOC web server (freely available at https://padloc.otago.ac.nz), allowing users to analyse whole genomes, metagenomic contigs, plasmids, phages and archaeal viruses. The web server includes a more than 5-fold increase in defence system types detected (since the first release) and expanded functionality enabling detection of CRISPR arrays and retron ncRNAs. Here, we provide user information such as input options, description of the multiple outputs, limitations and considerations for interpretation of the results, and guidance for subsequent analyses. The PADLOC web server also houses a precomputed database of the defence systems in > 230,000 RefSeq genomes. These data reveal two taxa, Campylobacterota and Spriochaetota, with unusual defence system diversity and abundance. Overall, the PADLOC web server provides a convenient and accessible resource for the detection of antiviral defence systems.

## Graphical Abstract

**Graphical Abstract:**
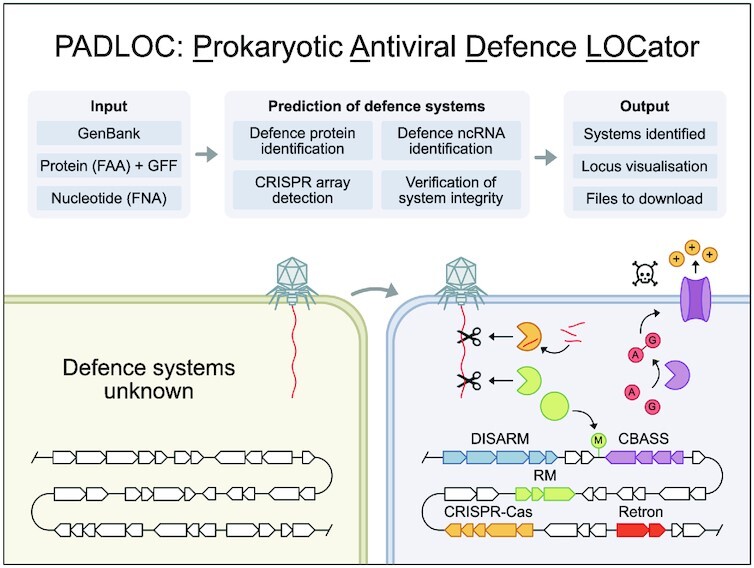
The PADLOC web server is a one-stop resource for the identification of antiviral defence systems in microbial genomes.

## INTRODUCTION

Diverse antiviral defence systems have evolved in bacteria and archaea that defend against infection by their viruses and mobile genetic elements. There are over 60 known broad families of defence systems, with more than 20 distinct system types discovered in the past five years (precise tallies are difficult since classification schemes, such as class, type and subtype, vary between families of systems and the mechanisms of many systems remain unknown) (1–5). As such, system discovery has greatly outpaced the development of tools that make use of these new insights. To provide widespread accessibility to known and newly discovered system types, and ensure consistency between system annotations, we recently developed the Prokaryotic Antiviral Defence Locator (PADLOC) tool as a framework to systematically identify antiviral defence systems (6). To simplify the use of PADLOC, we have developed the PADLOC web server, which expands the functionality of PADLOC, serves as a convenient and accessible interface for using the tool, and provides an extensive database of precomputed results – currently for more than 230,000 bacterial and archaeal genomes.

**Figure 1.**
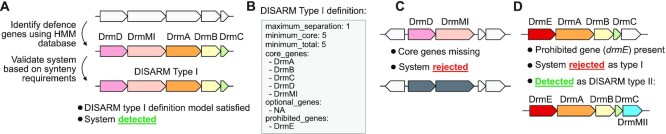
Defence system detection with PADLOC. (**A**) Genes encoding putative defence system proteins are identified using profile HMMs, then compared against the system definition models to determine whether a complete system is present. (**B**) For example, detection of a DISARM Type I system requires genes encoding all five core components (DrmA, DrmB, DrmC, DrmD, DrmMI) to be present. (**C**) If the minimum number of genes is not met, the system is not reported. (**D**) If any genes prohibited for a specific system definition are present (in the case of DISARM type I, *drmE* is prohibited), the system is rejected, but can instead be reported as a match to a different system definition—in this example as a type II DISARM (requiring genes encoding DrmA, DrmB, DrmC, DrmE and DrmMII).

Details regarding operation and benchmarking of the PADLOC tool itself have been described elsewhere (6). However, there are some important aspects of system detection that users of the PADLOC web server should understand when interpreting results. Genes encoding defence system proteins are identified by searching their protein sequences with a curated database of profile Hidden Markov Models (HMMs), currently representing > 700 families of defence-related proteins. Many of the protein families are represented by multiple HMMs (for example, there are currently 45 HMMs from various sources representing different Cas10 clades). Potential matches are filtered to remove low-scoring hits (based on E-value and coverage thresholds). PADLOC then uses a set of system definition models to determine whether the genetic synteny requirements are met for each possible system classification (Figure 1). This approach to multi-gene system identification has been applied successfully in the past for the detection of CRISPR-Cas (7,8) and protein secretion systems (9). In addition to detecting protein-coding genes, the PADLOC web server includes new functionality to detect CRISPR arrays and ncRNAs, such as retron msr-msd elements. Here, we discuss this added PADLOC functionality, the addition of many new systems to the database, limitations and important considerations for interpretation of the results, and guidance for subsequent analyses.

## MATERIALS AND METHODS

### Expansion of the PADLOC defence system database

At its core, the PADLOC web server is based on the PADLOC command line tool (https://github.com/padlocbio/padloc), with a curated database of profile HMMs, HMM scoring thresholds, and system models (https://github.com/padlocbio/padloc-db). Our current understanding of defence system genetics is the result of a collective scientific effort, and the HMMs and system models in the PADLOC database were built and curated using data from many sources. Construction of HMMs for the CBASS and Doron systems, plus several variant systems we discovered, are as previously described (6). We have since expanded the PADLOC web server database to contain > 180 system definitions, including > 3,500 profile HMMs. Where profile HMMs were made available by the authors of papers describing new defence system types (10–12), or databases and tools to detect subsets of systems (13–15), these HMMs were assigned PADLOC HMM accessions (e.g. PLDC12345) and added to the PADLOC database (the original HMM names were retained for traceability). Where multiple sequence alignments were available (16,17), we realigned the sequences using MUSCLE (18) and built HMMs with HMMER3 (19). For cases without HMMs or sequence alignments but a list of relevant proteins was available (8,20–39), we either used our sequence clustering and HMM generation pipeline described previously (6), or aligned the sequences with MUSCLE, manually curated the alignments to remove outlier sequences, then built HMMs with HMMER3. In the absence of supplied lists of homologs, we used example sequences from experimentally verified defence systems as seeds for BLAST searches, then aligned, curated and built HMMs, as above (22,40–69).

Where possible, the data source and appropriate reference for each HMM is listed in the PADLOC database HMM metadata file (hmm_meta.txt, available from the PADLOC database repository). We encourage PADLOC users to recognize the importance and value of these data used to build the PADLOC web server by citing the original sources. Models will continue to be added and updated periodically as more defence systems are discovered. We welcome and encourage submissions of new defence systems, including HMMs, multiple sequence alignments, or lists for relevant protein sequences or database accessions. Similarly, feedback to improve the sensitivity and specificity of PADLOC, updates to citation links, and suggestions to improve defence system and protein nomenclature will help ensure PADLOC remains a useful community resource.

### Detection of non-coding sequences

Since the command line PADLOC tool detects only protein-coding genes, yet many defence systems contain non-coding RNAs, we integrated detection of CRISPR arrays and retron-associated ncRNAs (msr-msd elements) into the web server. CRISPR arrays are detected with a customized version of CRISPRDetect (70) using the arguments: array_quality_score_cutoff 2.5; minimum_word_repeatation 3; word_length 11; minimum_no_of_repeats 3; repeat_length_cutoff 11; max_gap_between_crisprs 250. The resulting crispr.gff output file is supplied to PADLOC using the --crispr input option and the human-readable crispr.txt output file is made available for user download. Potential ncRNAs associated with retrons are identified by searching a database of msr-msd element covariance models (available from the PADLOC database repository), against each genome sequence using Infernal's cmsearch (71) with the arguments: Z 10; FZ 500. The Infernal output is filtered to only include hits passing the inclusion threshold (E-value = 0.01), then loaded into PADLOC using the --ncrna input option. In specific PADLOC system definition models (e.g. for retrons), ncRNAs are listed as ‘ncRNA’ in the core, accessory or prohibited gene lists, as required. As such, any identified ncRNAs contribute to the total required gene count for each relevant defence system.

### Precomputed RefSeq data and pseudogenes

For the precomputed PADLOC dataset (currently based on RefSeq v209 (72)), we used the [assembly]_genomic.fna, [assembly]_genomic.gff and [assembly]_protein.faa files for each genome assembly from the RefSeq FTP server. First, CRISPR arrays and retron-associated ncRNAs were identified by running CRISPRDetect and Infernal, respectively (as above), with [assembly]_genomic.fna as input. The resulting GFF-formatted CRISPRDetect outputs were saved for input to PADLOC and the more detailed, human-readable output files were loaded to the PADLOC web server for user download. Next, we pre-processed the [assembly]_genomic.gff and [assembly]_protein.faa files to allow increased detection of defence systems containing pseudogenes. The NCBI prokaryotic genome annotation pipeline (PGAP) identifies genes that contain frameshifts, nonsense stop codons, or appear otherwise incomplete, as pseudogenes (73). The coordinates of each pseudogene are reported in the [assembly]_genomic.gff, but the corresponding protein sequences are not included in the [assembly]_protein.faa. Since PADLOC relies on any potential defence system protein sequences to be present in the input file, pseudogenes belonging to defence systems would not normally be identified. The PGAP annotates each pseudogene with the accession of the full protein sequence used to infer the product of the pseudogene (e.g. the *Bacillus cereus* VD146 assembly GCF_000399425.1 has a pseudogene with locus tag IK1_RS32735 that is labelled as similar to the CRISPR-associated protein Cas4 of *Oceanobacillus massiliensis* WP_010649895.1). Therefore, we substituted the pseudogenes in each RefSeq genome with the sequence of their inferential protein (where available). Lastly, PADLOC was run using the pseudogene-corrected .gff and .faa inputs, plus the CRISPR array and ncRNA inputs (as above).

### Implementation

The core PADLOC tool is implemented in R, with some input handling using Bash and Python (primarily Biopython (74)). The PADLOC web server was built using the Django Framework (https://www.djangoproject.com). User jobs are identified by unique and anonymous job identifiers and are not accessible by other users. Users can access their results (tracked using cookies) until their browser cookies are cleared, the results are removed manually by the user, or until they expire (currently after 10 days). On the user side, anonymous job identifiers are replaced with the user-specific job name and output files downloaded are prefixed by the job name. When input files are uploaded to the server, a pre-processing script is used to detect the source format of the files and convert these to the default inputs for PADLOC (.gff and .faa with RefSeq formatting). For example, RAST formatted Genbank files are identified by the presence of the string ‘rasttk’, next the ‘db_xref’ field is changed to ‘locus_tag’, finally Biopython is used to output PADLOC-compatible input files. PADLOC also contains a ‘--fix-prodigal’ option to natively parse Prodigal-formatted .gff and .faa file pairs, which the pre-processing script detects by searching for the string ‘Prodigal’ in the uploaded .gff file. Although the command line version of PADLOC includes a wrapper for gene-calling with Prodigal (allowing input of unannotated nucleotide sequences), the web server runs Prodigal during the pre-processing stage, allowing different gene-calling settings to be used for inputs > 100 kb, versus shorter sequences (see the Prodigal documentation of an explanation of the rationale behind this). For users wanting to analyse unannotated plasmid sequences, we recommend including the host genome sequence within a multi-fasta input file before uploading to the PADLOC web server (to improve the quality of gene predictions with Prodigal). Once PADLOC is run, the output is passed to a post-processing script that reformats and outputs the data in a custom machine-readable format that is used to generate an interactive genome annotation display (produced using d3.js (https://d3js.org/)) on the corresponding user-job result page.

## RESULTS

### Input and file handling

**Figure 2.**
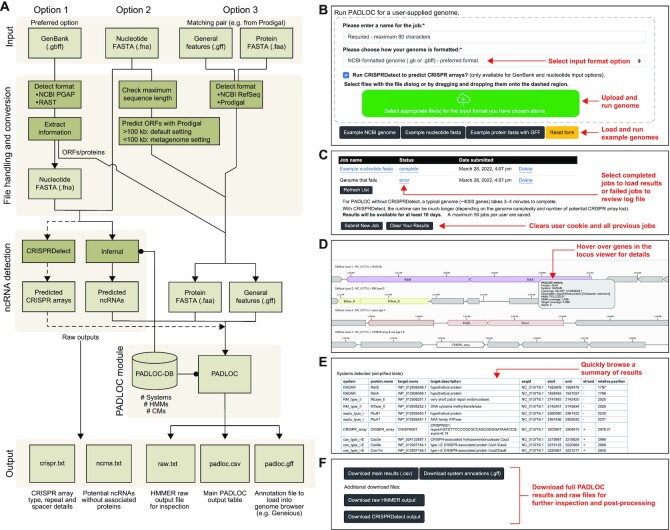
The PADLOC web server pipeline and results. (**A**) The PADLOC web server handles the pre-processing of user input to allow for additional input types and the identification of CRISPR arrays and ncRNAs. (**B**) Users can upload their own genomes or run an example genome through the ‘Run PADLOC’ page. (**C**) User jobs are listed on the ‘My Results’ page. Completed jobs link to their individual results pages, failed jobs link to a log file that provides information on why the genome could not be analysed. (**D**) The individual results pages display a locus viewer, which shows each identified defence system in the context of its surrounding genes. (**E**) A summarised version of the results is displayed under the locus viewer for an initial inspection of the systems identified. (**F**) Users can download the full PADLOC output and other raw files from the bottom of the page.

Users can analyse archaea, bacteria, metagenome, phage, archaeal virus and plasmid genome files from the ‘Run PADLOC’ page. The PADLOC web server accepts GenBank flat files, nucleotide FASTA, or paired amino acid FASTA and general feature format (GFF3) files as input (Figure 2A). If a GenBank file is provided, nucleotide, protein, and feature information (e.g. gene locations) is extracted using Biopython. If a nucleotide FASTA file is provided, Prodigal (75) is used to predict open reading frames and produce a protein FASTA and GFF3 file. For the best quality results, it is recommended that users supply a GenBank file or amino acid FASTA and GFF3 file where coding sequences have already been called and verified (e.g. with Prodigal or Prokka). Users may wish to download the example genome files to see the expected formatting for each file type (Figure 2B). When nucleotide information is provided, either through a GenBank or nucleotide FASTA file, Infernal (71) is used to detect the ncRNA components of retrons. Users also have the option to run CRISPRDetect (70) to predict CRISPR arrays. This additional information helps to verify and enhance the quality of defence system detection. All input files are passed to the core PADLOC module, which then detects the defence systems specified in the PADLOC database (a current list of systems is provided on the web server). The expected processing time for a typical genome encoding ∼5,000 proteins is less than five minutes. Including the CRISPRDetect option can substantially increase the run time in some cases, but usually only adds a couple of minutes.

### Output and interpretation

Each job submitted by a user is listed on the ‘My Results’ page (Figure 2C), which includes details of the job status and links to completed jobs. If the job failed, a link is provided to download the corresponding log file. The most common reason for a failed job is incorrect input formatting. For successful jobs, individual result pages contain information about interpreting the output, an interactive view of the locus structure of each detected defence system (Figure 2D), a summary table of systems detected (Figure 2E), and options to download the output files (Figure 2F). The main PADLOC output file (.csv format) lists all systems identified, with one gene per row. The file contains information regarding the type of system detected, the proteins present, their location in the genome, and details about the confidence of detection. The most important values to consider when evaluating detection confidence are the full sequence and domain E-values (full.seq.E.value and domain.iE.value), and the target and HMM coverages (target.coverage and hmm.coverage). Usually, hits with large E-values (indicating low statistical significance) and low coverages should be treated with caution. In general, multi-gene defence systems are detected with greater specificity than systems encoded by singe genes.

### Considerations and limitations

As with any computational approach to inferring gene function, there are several potential limitations that users should consider when interpreting PADLOC web server outputs. Many defence proteins contain ubiquitous domains and their HMMs are more likely to detect spurious hits. For example, PtuA (Septu), several retron proteins and Old nucleases contain similar ATPase domains (11). As a result, PADLOC sometimes reports overlapping system classifications (typically less than 1% of results), which should be resolved via subjective evaluation of the reported scoring parameters and genetic context. For multi-gene systems, the synteny requirements resolve many ambiguities and increase the confidence of system classification (due to the reduced probability of two adjacent false-positive hits). By contrast, identification of single-gene systems is more challenging and requires trade-offs with the HMM scoring cut-offs (E-value and HMM/target coverage thresholds) to achieve an acceptable balance between sensitivity versus specificity. As such, false positive and negative results are inevitably more frequent for single gene systems. In general, the PADLOC scoring thresholds are set more toward sensitivity, with the intention that users interested in further study of identified potential defence system homologs will undertake additional analyses.

**Figure 3.**
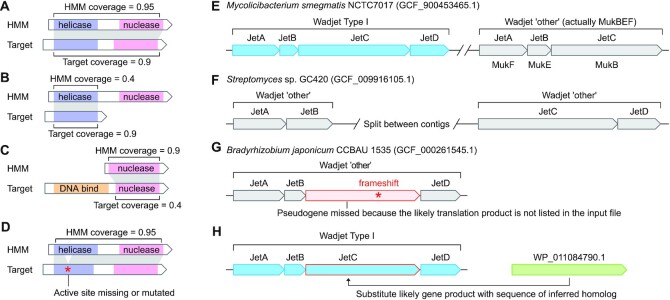
Considerations and limitations of defence system detection using profile HMMs and synteny criteria. (**A**) Likely hits to defence protein homologs have high alignment coverage between the HMM and target protein, including for multi-domain proteins, as the PADLOC HMMs were typically built from whole proteins rather than individual domains. (**B**) Users should be wary of cases where only part of the HMM aligns to the target protein, which may lack a domain important for function of defence system homologs. (**C**) Conversely, some defence protein domains have similarity to domains found within non-defence proteins, which can result in the PADLOC HMMs matching only part of the target protein. However, these cases might also represent defence system fusion proteins, or divergent homologs. (**D**) Where possible, users should follow up by also verifying the presence of expected active site residues and motifs important for domain fold and function. (**E**) Some defence systems are similar to non-defence molecular systems, such as the similarity between Wadjet and Muk systems. This is typically not an issue with multi-gene systems where all genes are present, but some [system]_other models may detect such cases where some genes are allowed to be absent. (**F**) Several [system]_other models allow the detection of fragmented multi-gene systems, which can be reconstructed manually after reviewing the results. (**G**) Pseudogenes within multi-gene systems are not detected by the default PADLOC workflow, so users should check [system]_other models for the potential presence of additional genes. In this example, a frameshift within *jetC* means the Wadjet system criteria are not fulfilled (because JetC was not detected) (**H**) In the precomputed PADLOC RefSeq dataset, pseudogenes were substituted with the protein homologs inferred by the PGAP pipeline, allowing the above example to be classified as a Wadjet Type I system. Pseudogenes are indicated by a red outline in the locus viewer and the prefix ‘pseudo_sub’ in the ‘target.name’ column of the output.

As a first step to curating PADLOC results, inspection of the HMM and target alignment coverage scores can reveal potential false-positive classifications (Figure 3A–C). In some cases, very similar proteins differ in function due to the presence or absence of enzymatic sites or functional motifs (Figure 3D). We encourage users to explore subsequent domain-based analyses of defence system proteins using tools such as HHpred (76) to identify protein domains with more granularity. We have also found structure prediction tools such as AlphaFold2 (77) and ColabFold (78), useful in identifying domain folds and boundaries. Once demarcated, predicted domain structures can be searched against protein structure databases such as the PDB (79) or AlphaFold-based databases, using tools like DALI (80) or Foldseek (81). In many cases, this structure-based approach reveals homologs with characterised active sites or functional motifs, which can aid in discrimination between defence system proteins and similar non-orthologous proteins. Users may also find it informative to compare their PADLOC results with DefenseFinder, another tool recently developed for defence system identification (82,83).

The similarity of several defence systems to other molecular systems inevitably leads to a background rate of false-positive system identifications. For example, Wadjet systems are similar to Muk structural maintenance of chromosomes (SMC) systems (21,84,85) (Figure 3E). The canonical Wadjet system comprises four proteins JetABCD, where JetA, JetB, and JetC share similarity with MukF, MukE, and MukB, respectively. The PADLOC Wadjet system definition requires the full JetABCD set to be present, whereas the MukFEB cases are reported as part of Wadjet ‘other’ systems. Several ‘[system]_other’ models (which generally require only two components of a system to be co-localised) are run alongside the stricter canonical system definitions, to enable identification of systems that might otherwise be overlooked due to their being split by contig boundaries (particularly in metagenomes) (Figure 3F), fragmented by multiple intervening genes (e.g. due to MGE insertions), genes missing due to sequencing, assembly or gene-calling errors, mutations, or high sequence divergence of some defence proteins (Figure 3G). For the precomputed RefSeq data, we substituted pseudogene products with similar full-length protein sequences, which allows higher-confidence assignment of the example system as Wadjet type I (Figure 3H). The identification of defence system pseudogenes will also be helpful for studies of defence system evolution and turnover. For some systems, such as the Dnd and Pbe phosphorothioation systems, several genes are relatively short (including *dndE, pbeB/D*) and are often missed by gene prediction tools (33,35). The ‘PT_other’ model will detect the remaining genes and users should then manually check for short coding sequences in the vicinity of the expected location of any absent genes. Lastly, several system definition models specify ‘optional’ genes (e.g. ‘cas_associated’ proteins) that are not necessarily functionally associated with the system. In some cases, these proteins may be uncharacterised independent *bona fide* defence systems, or might have non-defence functions. To guide users in interpreting results for ‘other’ models and resolving ambiguities, each ‘Results’ page on the PADLOC web server contains a list of known potential ambiguities.

### The current snapshot of antiviral defence systems

**Figure 4:**
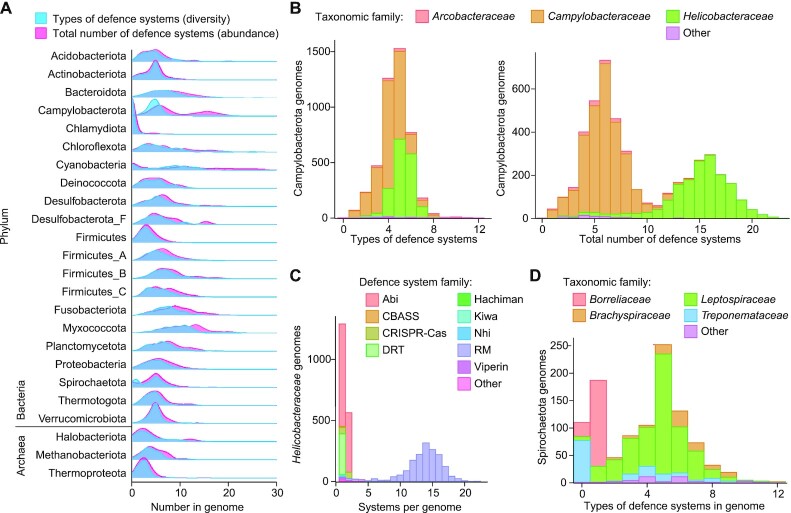
Example analyses of PADLOC data reveal lineage-specific differences in defence system diversity and abundance. (**A**) Overview of the defence system diversity (the number of unique types of defence systems within a host genome) and abundance (total number of defence systems within a host genome), separated by phyla (per the GTDB taxonomy (91)). Only phyla with more than 50 genomes are displayed. (**B**) A closer look at defence system diversity and abundance within the Campylobacterota phylum. (**C**) A breakdown of the abundance of different defence system types within the *Helicobacteraceae* family of Campylobacterota. Defence system types occurring in less than five genomes are grouped under ‘Other types’. (**D**) Defence system diversity within Spriochaetota, revealing low defence diversity within *Borreliaceae*. The *Treponemataceae* lacking defence systems (types = 0) in this dataset are comprised entirely of *Treponema pallidum*.

To provide a current and comprehensive quantitative view of the defence systems in bacteria and archaea, we used PADLOC to search all RefSeq v209 Bacteria and Archaea genomes. These results are available for browsing on the PADLOC web server under the ‘RefSeq results’ page and will be updated periodically with new RefSeq versions and as new defence systems are discovered. Overall, the distribution of different defence systems across different bacteria and archaea is highly varied (Supplementary Figure S1). It should be noted that this analysis includes assemblies of varied completeness, and systems may be underrepresented in taxa with incomplete genomes. Clear differences in the diversity and abundance of defence systems were apparent between phyla (Figure 4A), with several notable outliers including Chlamydiota (very few known defence systems) and Cyanobacteria (many types of defence systems in high abundance). Campylobacterota had a significant bimodal distribution of defence system abundance (Hartigan's dip test (86), *P* < 0.001), which relates to the *Helicobacteraceae* relying on a remarkably large number of restriction modification systems in each strain (Figure 4B,C) (87,88). Another interesting phyla was Spirochaetota, which had a significant bimodal distribution of defence system diversity due to *Borreliaceae* having very few types of defence systems (Hartigan's dip test (86), *P* < 0.001) (Figure 4D). Almost all *Borreliaceae* are tick-borne pathogens (89) with characteristically small genomes typical of obligate host-associated pathogenic bacteria (90). Similarly, *Treponema pallidum* (family *Treponemataceae*) are host-associated pathogens with small genomes and lack known defence systems (Figure 4D). It remains to be resolved whether the low defence diversity is driven by genome reduction and due to less exposure to phages and MGEs than free-living relatives. Overall, these examples illustrate that the PADLOC web server can be used to interrogate hypotheses such as these and to then identify candidate taxa and strains in which predictions can be experimentally tested.

## DISCUSSION

To address the lack of capable and accessible tools for comprehensive defence system identification, we developed the PADLOC web server. Here, we have described the current state of PADLOC and important information regarding usage of the web server and interpretation of the output. PADLOC will continue to evolve as new defence systems are discovered and additional biological insight allows us to fine-tune the parameters of identification. Evaluating the accuracy of detection and adjusting these thresholds accordingly is difficult when only a few experimentally verified examples are available, as is currently the case with most defence systems. PADLOC provides a key initial step for further improvement, by facilitating the identification of many putative systems that can be followed up by functional investigation. Feedback and contribution to PADLOC and the web server is encouraged via the GitHub repository (https://github.com/padlocbio/padloc/issues), including but not limited to the addition of systems and HMMs, suggestions for adjusting thresholds or system classifications and nomenclature, and reporting bugs.

The comprehensive identification of many systems made possible with PADLOC also opens avenues for investigating many interesting biological questions. For example, many putative defence system genes are annotated as pseudogenes. Although pseudogenes can arise from sequencing or assembly errors, they might also include the remnants of defence systems that have become inactivated through mutation. Investigation of these remnants could provide insight into the ancestry and evolution of defence system arsenals that would otherwise remain undetected. In addition, our broad taxonomic analyses revealed notable differences in the diversity and abundance of defence systems in many taxa, raising the question of what factors drive the requirement for more defence against phages, and whether differences are driven by ecological factors such as phage diversity and encounter rates (reviewed in 92). Recent studies have also identified interplay between defence systems, with synergistic or antagonistic effects (42,93,94) and as more types of defence systems are discovered, our understanding of compatibility between systems needs to be revisited. The PADLOC web server provides a convenient and accessible platform to detect suitable candidate strains for experimental work to resolve these outstanding questions.

## DATA AVAILABILITY

The PADLOC web server is freely available at https://padloc.otago.ac.nz. This website is open to all users and there is no login requirement. Source code and documentation for installing and running PADLOC locally are freely available from the PADLOC GitHub repository (https://github.com/padlocbio/padloc). The HMMs and system models used by the PADLOC web server are available from the PADLOC database repository (https://github.com/padlocbio/padloc-db).

## Supplementary Material

gkac400_Supplemental_File
