# Supplementary material for: PADLOC: a web server for the identification of antiviral defence systems in microbial genomes
Source: Nucleic Acids Res. 2022 May 25;50(W1):W541–50. doi: 10.1093/nar/gkac400 (PMC9252829; doi:10.1093/nar/gkac400)

PADLOC: a web server for the identification of antiviral defence systems in microbial genomes

Leighton J. Payne[1], Sean Meaden[2], Mario R. Mestre[3], Chris Palmer[4], Nicolás Toro[5], Peter C. Fineran[1,6,7,8], Simon A. Jackson[1,6,7,8*]

Supplementary information

Figure S1. Overall defence system distribution. All genomes from RefSeq v209 Archaea and Bacteria were searched with PADLOC. The values in the boxes represent, for each phylum, the average percentage (rounded up to nearest 1%) of genomes in each species of that phylum encoding a system, grouped using GTDB taxonomy; system prevalence is weighted in this way to limit biases in phyla that contain many closely related genomes of the same species. The colouring in each box provides a visual representation of these values.

Figure S1. Overall defence system distribution.

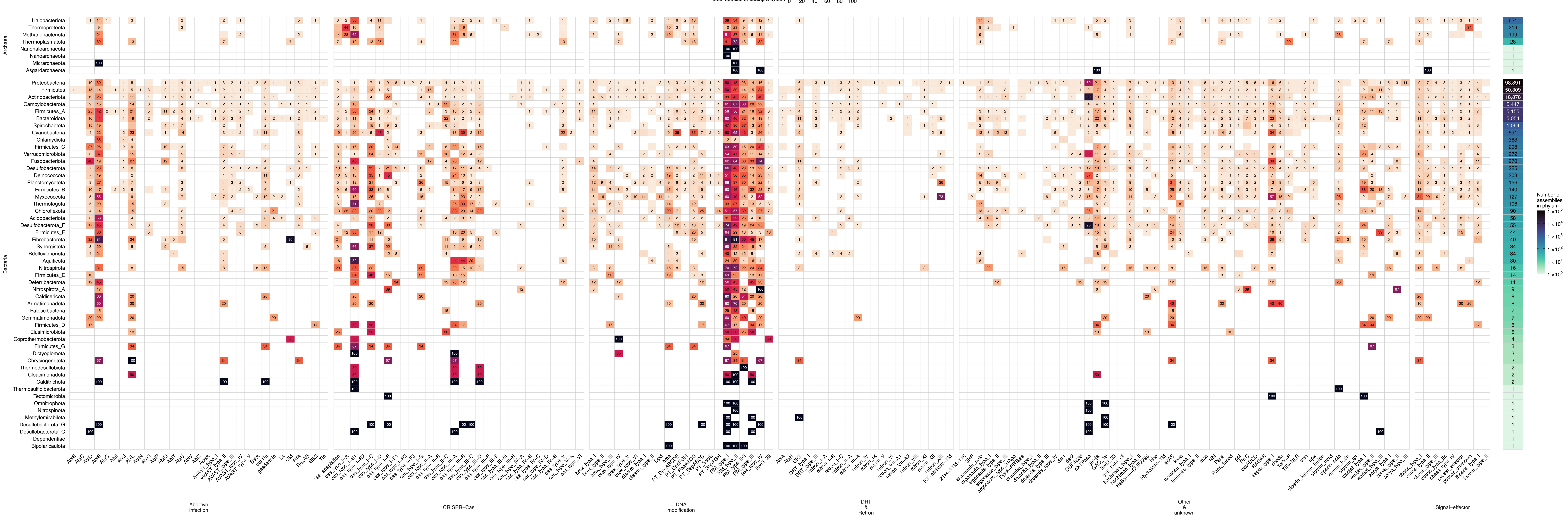

Supplement: gkac400_Supplemental_File [file gkac400_supplemental_file.pdf]
